# Evaluation of Spoken Dialogue Technology for Real-Time Health Data Collection

**DOI:** 10.2196/jmir.8.4.e30

**Published:** 2006-12-11

**Authors:** Esther Levin, Alex Levin

**Affiliations:** ^2^Spacegate IncLivingstonNJUSA; ^1^Department of Computer ScienceCity College of New YorkNew YorkNYUSA

**Keywords:** Human factors, ecological momentary assessment, data collection, voice recognition

## Abstract

**Background:**

A real-time assessment of patients’ experiences is an important methodology for studies in health care, quality of life, behavioral sciences, and new drug and treatment development. Ecological momentary assessment is a methodology that allows for real-time assessment of experience and behavior in a subject’s natural environment. Recently, electronic data collection techniques have been introduced, including systems utilizing interactive voice response.

**Objective:**

The objective of this project was evaluation of spoken dialogue methodology for real-time data collection of information from patients for health, behavioral, and lifestyle studies and monitoring. While the management of the data collection process was Internet-based, this additional eHealth communication channel was based on over-the-phone natural language conversation with a dialogue system utilizing automated speech recognition technology. For this study we implemented a dialogue system for patients’ assessment and monitoring of chronic pain.

**Methods:**

Experimental evaluation of usability of the Pain Monitoring Voice Diary was performed with 24 volunteers. The volunteers were asked to contribute 10 sessions with the system over a period of 2 weeks; in practice, the number of sessions per subject ranged from 1 to 20. The subjects were asked to either relate to pain episodes in their past while answering the system’s questions, or use as a guidance one of nine provided medical scenarios compiled by a pain specialist, ranging from migraines and back pain to post-surgical pain (knee injury) and cancer- and chemotherapy-related afflictions.

**Results:**

From 24 volunteers, we collected a total of 177 dialogue sessions: 171 sessions were completed, while the caller hung up in the other 6 sessions. There were a total of 2437 dialogue turns, where a dialogue turn corresponds to one system prompt and one user utterance. The data capture rate, measuring the percentage of slots filled automatically, was 98%, while the other 2% were flagged for transcription. Among the utterances sent to transcription, where the user had opted for the “none of those” option, 70% corresponded to the “type of pain” slot, 20% to the “symptoms” slot, and 10% to the “body part” slot, indicating that those are the grammars with the highest out-of-vocabulary rate.

**Conclusions:**

The results of this feasibility study indicated that desired accuracy of data can be achieved with a high degree of automation (98% in the study) and that the users were indeed capable of utilizing the flexible interface, the sessions becoming more and more efficient as users’ experience increased, both in terms of session duration and avoidance of troublesome dialogue situations.

## Introduction

Use of questionnaires is an essential method of data collection, especially in studies of health care, quality of life, behavioral sciences, and new drug and treatment development. A real-time assessment of experience and behavior in the patient’s natural environment is an important parameter that provides feedback and input to the health professional, researcher, or pharmaceutical company about the effects of treatment and/or the patient’s quality of life. Very often the research or study findings are based significantly or completely on questionnaire responses. While designing valid questionnaires is an art, the tools and methods of data collection are not less important and often can influence the research outcome. Ecological momentary assessment (EMA) is a methodology that allows for real-time assessment of experience and behavior in a subject’s natural environment. The methodology has evolved from the behavioral sciences [1-6] and enables the gathering of meta-data on patient compliance, as well as the measurement and improvement of compliance.

Traditional EMA data collection methods vary from paper-based diaries and reports to video/audio recordings and to human observation. However, doubts have been cast on the validity of the data collected through paper-based methods of self-report, notably from a recent study that demonstrated that most of the paper diary entries by patients (79%) were falsified [7]. Recently, electronic data collection techniques have been introduced, including personal digital assistants (PDAs), the Internet [8], and cellular phones utilizing interactive voice response (IVR). These methodologies enable collection of meta-data on the respondent’s compliance and use of such data to measure and improve compliance. While PDAs and Internet collection methods demonstrate clear advantages over paper forms, they also have certain limitations, including use of uncommon devices requiring participant training, limited availability, and extensive data management and programming costs. In addition, PDA methods require in-person contact to download data and change batteries. In IVR-based data collection participants use phones to call the system and answer the question posed by the system by pushing keys on the phone. Collins et al have reported on the compatibility of phone-based IVR data collection and its improvement over paper-based and PDA-based systems [9].

Spoken language dialogue system for data collection [10-13] is based on automatic speech recognition and, similarly to IVR, is a phone-based approach. It extends the IVR approach by allowing the subject to communicate with the system using a natural spoken language as an input modality. A challenge such a system faces is maintaining adequately high accuracy of the captured data while guaranteeing a satisfactory user experience. In particular, since the subjects conduct dialogues with the system on a regular basis, adequate dialogue design should provide a flexible level of user support to accommodate both novice callers and experienced callers: for the experienced caller, the system needs to provide short and effective call flow without making the caller hear long and tedious prompts; for the novice caller, the system needs to provide enough information and help to guarantee question understanding and successful session completion.

In this paper we describe a design for spoken language dialogue that takes into account the specificities of the data collection task. We first present spoken dialogue technology and its potential applicability to data capture task. We then describe a Pain Monitoring Diary dialogue design and how it addresses specific requirements of data capture task. We then report the results of a feasibility study.

## Methods

### Data Capture via Spoken Dialogue System

**Figure 1:**
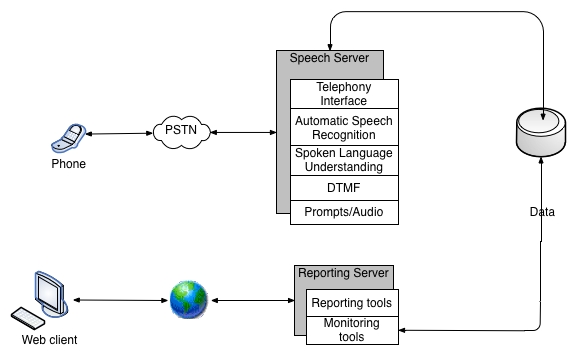
Block diagram of the data collection system; PSTN: public switched telephone network; DTMF: dual tone multi-frequency

Figure 1 presents the block diagram of a spoken dialogue system. The system has two interfaces: the first for the subjects and the second for the health care providers or researchers gathering the data. The subject communicates with the system through phone dialogue. A recording of such an interaction is included in Multimedia Appendix 1 and its transcription is shown in Textbox 1.

Each data collection session is scheduled according to a protocol and is initiated either by the system or by a subject. During the phone session, the utterances spoken by the subject are captured by the speech server. The speech server invokes automated speech recognition (ASR) and spoken language understanding modules to convert spoken utterances to textual semantic representation of subject’s inputs and then stores them in the database. The reporting server can then use this data in real time to generate a report about the session that can be viewed by a health care provider or a researcher several seconds after the session ends. The reporting server works in a push or a pull mode, or both. In the pull mode, the reports can be viewed over the Internet on demand; in the push mode, the reporting server sends notifications and alerts (via email, cell phone, or pager) to the health care provider about the availability of the new report. Table 1 shows a report automatically generated by the system at the end of the session presented in Textbox 1.

**Transcription of a sample dialogue**

**System:** Your PIN, please.

**Caller:** ****

**System:** OK, *****, let’s begin. Are you in pain now?

**Caller:** Yes.

**System:** Where does it hurt? [pause] For example, your head, stomach...

**Caller:** My left shoulder.

**System:** Was that your left shoulder?

**Caller:** Yes.

**System:** Please rate your pain on a scale of 0 to 10, with 0 being no pain and 10 being the worst possible pain.

**Caller:** It is 7.

**System:** Was that 7?

**Caller:** Yeah.

**System:** How does the pain feel?

**Caller:** I don’t know.

**System:** Okay, here is the help information. At this point, I need to find out the type of your pain. Please choose carefully a word from the following list that best describes your pain and just say it. If nothing matches, please say “none of those.” Here is the list: aching [pause], burning [pause], dull…

**Caller:** It feels sharp.

**System:** Was that “sharp?”

**Caller:** Yeah.

**System:** Is the pain constant or does it come and go?

**Caller:** Comes and goes.

**System:** Do you have any other symptoms?

**Caller:** Yeah.

**System:** Please tell me your most disturbing symptom.

**Caller:** I have fever.

**System:** I heard “fever.” Is that right?

**Caller:** Yes.

**System:** Would you like to leave a message for the staff?

**Caller:** No.

**System:** Thanks for your cooperation. I’ve got all the information that I need. I’ll call you again in 4 hours. Goodbye.

There are at least four advantages of using spoken dialogue technology for data collection compared to traditional methods using pen and pencil. First, speech is a natural modality of interaction for humans, and the input device—the phone—is user friendly and ubiquitous and no special training is required for its use (as opposed to PDAs or computers). Second, compliance is monitored automatically: the calls can be initiated by the system following a prescribed protocol, and the system can report any noncompliance to the trial administrator in real time. Third, spoken automated dialogue reaches much beyond voice-enabled static paper questionnaires: possible answers are not limited by the number of check-boxes to fit on a piece of paper; question selection can be done dynamically based on previous answers; and personalization of both content and style based on the patient’s history is possible. Fourth, the ability to transform the captured data into real-time reports and further interface the information with other clinical or back-office systems and databases provides an unparalleled opportunity to enhance patient feedback and monitoring. Overall, an ASR-based system offers the health care provider an extensive and practical tool to facilitate efficient and convenient patient communications, which saves time while increasing quality of care.

**Table 1:** Automatically generated report for the dialogue in Textbox 1

	**Captured Value**	**Confirmed (yes/no)**	**Confidence Score**
PIN	****	no	66
Are you in pain?	yes	no	80
Pain location	left shoulder	yes	86
Pain intensity	7	yes	88
Pain type	sharp	yes	88
Pain constant?	pain comes and goes	no	47
Symptoms	fever	yes	86
Message	none	no	78

### Pain Monitoring Diary Application

**Figure 2:**
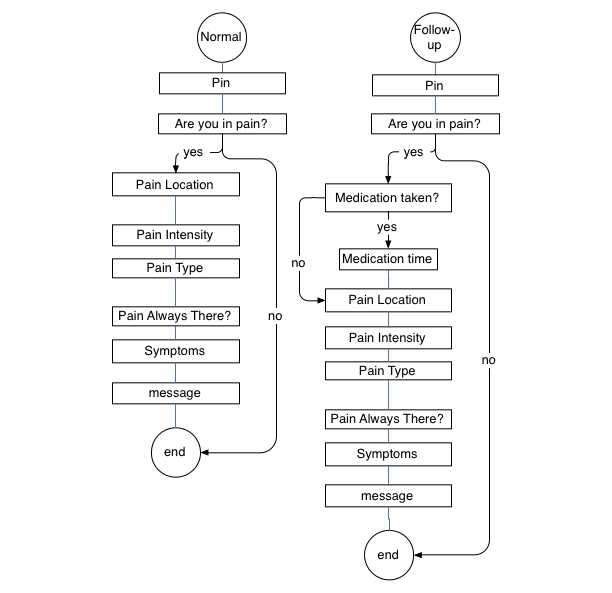
Schematic illustration of call flow for the Pain Monitoring Diary in normal and follow-up modes

For this study, we implemented a dialogue system for chronic pain patients’ assessment and monitoring. Pain assessment is an application for which well-established standard questionnaires [14,15] are available, and the vocabulary for potential answers can be established from the medical literature. Figure 2 shows dialogue flow for the Pain Monitoring Diary. The dialogue flow is represented as a series of dialogue units, where each unit comprises several caller-system exchanges designed to elicit one piece of information from the caller to fill a slot in the session report. A slot is, for example, pain location, pain intensity, pain type. Please note the correspondence between the dialogue units in Figure 2 and the dialogue session (Textbox 1) and its report (Table 1).

### Dialogue Design

The characteristics and requirements of data capture tasks are different than those for other applications of spoken dialogue technology [16]. Successful dialogue design needs to take the following specificities of this task into account.

First, data validity, accuracy, and integrity in this application are very important since the penalty for an erroneously filed final session report can be very high. Since the ASR technology is not perfect, the design has to take into account the possibility of speech recognition errors and improve the overall accuracy using dialogue actions such as re-prompts, confirmations, error handling, and, if necessary, recording and flagging the unrecognized utterances for later transcription.

Second, subjects call the system repeatedly according to the study protocol and identify themselves at the beginning of the session. This provides an opportunity to use the knowledge accumulated across sessions for personalization.

Third, the system should accommodate both novice callers (in the beginning of the trial) and experienced callers (those who completed several sessions). For the experienced caller, the system needs to provide a short and effective call flow, without making the caller hear long and tedious prompts. For the novice caller, the system needs to provide enough information and help to guarantee question understanding and successful session completion.

Fourth, the subjects participating in data collection are enrolled through a personal face-to-face interview during which they receive relevant information about the trial and guidance on the process of data collection. In the same opportunity, the participants can receive some training, explanation, and possibly a demonstration on how to use the spoken dialogue system.

Based on these considerations, our goals in the design of the dialogue system were controlling the accuracy of the captured data while providing a flexible and adequate level of user support allowing efficient communication for experienced users and sufficient support for novices.

### Controlling Captured Data Accuracy

We designed the system to take into account the known limitations of ASR technology and to be able to ensure overall high accuracy of data capture and session completion rate. In general, the most important parameter that determines the accuracy of speech recognition is the size and the complexity of the grammar that is being used for recognition of the current utterance. The grammar in speech recognition describes (as text) the set of all the possible sentences that can be recognized by the system. For example, the simplest grammar that can be used in yes/no recognition will contain only two sentences: {“yes,” “no”}. During speech recognition, current user utterance is matched to every sentence in the grammar, and the recognition result is given as the best matching sentence within the grammar, together with a recognition score that measures the quality of the match. If the quality of the match is not good enough, the recognizer will output a “rejection.” What happens when an out-of-vocabulary utterance (utterance not covered by the grammar) is spoken by the user? The recognizer is still trying to match it to the set of sentences described by the grammar and will output the best match, or rejection. Since the grammar does not contain the spoken sentence, the best match of the recognizer in the out-of-vocabulary case is always erroneous; therefore, to improve the accuracy of the grammar, we need to expand it to cover as many possible sentences that the user can utter. For example, in the yes/no grammar, we incorporated many variations of “yes” and “no,” such as “yeh,” “sure,” and “nope.” Another way to control the accuracy is to improve the rejection mechanism to guarantee that out-of-vocabulary utterances will be rejected.

Based on these considerations, we deployed the following methodology in the system design described below: 1) improving rejection mechanisms for confirmation and other grammars, 2) using confirmations as the way to control the larger grammar’s accuracy, and 3) using recordings to capture the unexpected and problematic spoken responses.

#### Improved Rejection Mechanisms for Confirmation and Other Grammars

We incorporated a garbage model in the yes/no grammar used for confirmations in our application. The garbage model was designed to match out-of-vocabulary utterances [17,18], specifically the corrections users are frequently providing instead of a negative confirmation (ie, those utterances that do not represent “yes” or “no” answers), for example

System prompt: Was that your left shoulder?

User: No, right shoulder.

(See also Multimedia Appendix 2.)

We used rejection criteria based on a combination of recognition score and garbage model scoring to control the overall accuracy of this grammar. In the case when, for a given spoken utterance, the recognizer outputs a hypothesis that is a part of a garbage model, this utterance is rejected. Also, if the hypothesis is not part of a garbage model, but has a low recognition score, it is rejected as well.

#### Using Confirmations as the Way to Control the Larger Grammar’s Accuracy

The grammars that are substantially larger than yes/no are also those for which we can expect more ASR errors and out-of-vocabulary utterances. Those are grammars like the body part grammar or the symptoms grammar when, without substantial data collection, we cannot accurately predict all possible ways the users will answer the questions “Where does it hurt?” or “What’s your most disturbing symptom?” For such grammars, we use the confirmation mechanism to control the overall accuracy of the data we capture. The result is considered captured only if the user answers “yes” to the confirmation question, reducing the error rate for the dialogue units with larger grammars to the level of yes/no grammar.

#### Using Recording to Capture the Unexpected and Problematic Spoken Responses

In almost all applications of ASR, especially in spontaneous speech tasks, the recognizer vocabulary cannot cover all occurring words. There is always a significant amount of out-of-vocabulary words even when the vocabulary size is very large. For example, an out-of-vocabulary input can occur when the user is trying to answer the question “Where does it hurt?” with a word or a phrase that is not covered by body part grammar. Since, by definition, out-of-vocabulary input cannot be recognized correctly, it can lead to either a rejection by the recognizer or to misrecognition to be followed (presumably) by a negative confirmation. In both cases, as will be explained in the next section, the system will detect speech recognition failure, will describe in the next prompt what the in-vocabulary choices are, and will offer the user to say a key phrase like “none of those,” indicating that the choices offered do not adequately describe the user’s intended input. When the user chooses the “none of those” option, the system will record the user’s input and flag it as “transcription is needed” for later processing.

System prompt: Was that your left shoulder?

User: No.

System prompt: Sorry about that. Let’s try it this way. Please choose carefully a body part from the following list that best describes the location of your pain, and just say it. If none of the locations match, please say “none of those.” Here is the list: abdomen [pause], ankles…

User (interrupts): None of those.

System prompt: OK. Let me just record your answer. Please describe the location of your pain in your own words.

User: [description of pain location]

System prompt (after recording is finished): Thanks, I got that. Let’s move on.

(See also Multimedia Appendix 3.)

The same mechanism of fall-back to recording instead of recognition is used after several repeated recognition failures, like in a case when the user does not chose the “none-of-those” option, while repeating the out-of-vocabulary input.

In the following example, the user is trying to answer the question about type of pain with an unexpected out-of-vocabulary input.

System prompt (following negative confirmation): Sorry about that. Let’s try one more time. Please choose carefully a word from a following list that best describes your pain and just say it. If nothing matches, please say “none of those.” Here is the list: aching [pause], burning [pause], dull…

User (interrupts with out-of-vocabulary input): I have high fever.

System prompt: Was that “deep?”

User: No.

System prompt: Sorry, I am having trouble. Let me just record your answer. Please describe how your pain feels in your own words.

User: I am burning with fever.

System prompt (after recording is finished): Thanks, I got that. Let’s move on [pause]. Is the pain constant…?

(See also Multimedia Appendix 4.)

### Flexible Level of User Support

The flexible level of user support that is intended to satisfy both the novice and the experienced user is achieved by prompt design, context-sensitive help, detecting speech recognition failures, and dialogue personalization, described below.

#### Prompt Design

The system prompts are designed to provide an appropriate level of support to the user. For example, the initial prompt for the pain location dialogue unit is “Where does it hurt?” [pause]. For example, your head, stomach, or back? [pause]. Remember, if youdon’t know how to answer this question, just say ‘I need help.’” The pauses in this prompt are designed to encourage the experienced user to interrupt the prompt with the answer (most experienced users interrupt after the initial prompt), while providing more information (in this case, examples of possible answers) for the inexperienced user who hesitates to answer immediately. It also reminds the user to ask for help if it is still not clear what can be said as an answer. Multimedia Appendices 5, 6, and 7 contain the recordings illustrating the different user experiences with this prompt.

#### Context-Sensitive Help

Although participants may have received some training at their orientation session, it is unreasonable to expect them to retain this information for the whole duration of the trial, which can last for months. Therefore, for every question in the Pain Monitoring Voice Diary help information is provided upon the user’s request, describing and clarifying the current question, and, in some cases, enumerating the possible answers the caller can choose from while, in other cases, giving more examples of possible answers. For example, if the caller asks for help after the “Where does it hurt?” question, the system will provide a very elaborate help prompt that lists different body parts the user can say (pausing shortly after each one to encourage the user to interrupt if the user knows what to say). It also reminds the user about the “none of those” option:

Okay. Here is the help information. At this point I need to find out the part of your body that hurts the most. Please choose carefully a body part from the following list that best describes the location of your pain, and just say it. If none of them matches, please say “none of those.” Here is the list: abdomen [pause], ankles [pause], back [pause]...toes [pause]. Which one is it?

The information provided during these explicit requests for help closely follows the information the user received during the enrollment process. The recording in Multimedia Appendix 8 illustrates a case of help request.

#### Detecting Speech Recognition Failures

Even when the user has not asked for help explicitly, the dialogue is designed to detect the user’s repeated failures and provide more support. When the system experiences recognition problems, such as rejection or silence, it will re-prompt the user for the same question. The re-prompts are designed as an escalating list, providing increasingly more information and progressively constraining the user as more such errors are detected. For example, if the user’s utterance is rejected by the recognizer after the initial prompt “Where does it hurt? [pause]. For example, your head, stomach, or back? [pause]. Remember, if you don’t know how to answer this question, just say ‘I need help,’” the system will re-prompt for the same information with “I didn’t get that. Let’s try it this way. Please tell me the part of your body that hurts the most. Remember, you could always say ‘I need help.’” The second prompt skips the pauses and reminds the user to ask for help if needed, and it also clarifies the question (“body part that hurts the most”).

Another case when the system detects that something went wrong with speech recognition is when the user says “no” to a confirmation question:

System prompt: Was that your left shoulder?

User: No.

System prompt: Sorry about that. Let’s try it this way. Please choose carefully a body part from the following list that best describes the location of your pain, and just say it. If none of them matches, please say “none of those.” Here is the list: abdomen [pause]…. Which one is it?

(See also Multimedia Appendix 9.)

Since the user disconfirmed the recognized body part, the system detects a recognition problem and gives the user more information on how this question can be answered to minimize the out-of-vocabulary utterance rate.

### Dialogue Personalization

Data capture is a unique dialogue application since the users not only call the system many times during the trial, but they also identify themselves at the beginning of each session. This provides a system with an opportunity to personalize both the content of the current session (what are the data to be collected) as well as the style (how to ask for these data) based on the results of the previous sessions. As shown in Figure 2, in our system we took advantage of a larger inter-session context by designing two types of data collection sessions: normal and follow-up. The follow-up session type is deployed if the subject reported a high level of pain in the previous session. The follow-up session differs from the normal one not only by the additional questions the patient is asked, such as if and when the subject took the medication, but also by the format of the questions. If, in the previous session, the subject reported pain in the left shoulder, in the follow-up session the question will be “Is the pain still in your left shoulder?” (see Multimedia Appendix 10). This format of “reminding” prompts was used for pain location and pain type dialogue units, and it was designed to possibly shorten the dialogues and also provide the user comfort and a feeling of continuity in using the system. Textbox 2 presents a transcription of a follow-up session (it is the follow-up for the normal session shown in Textbox 1) and Multimedia Appendix 11 contains its recording.  Table 2 describes an automatically generated report for this follow-up session.

**Transcription of a follow-up dialogue**

**System:** Your PIN, please.

Caller: ****

**System:** Hi, Esther, This is your follow-up call…. Are you still having pain?

**Caller:** Yeh.

**System:** Did you take your pain medication today?

**Caller:** Yes.

**System:** What time did you take the medication?

**Caller:** Two hours ago.

**System:** Was that around 9:45 am?

**Caller:** Yeh.

**System:** Did the medicine diminish your pain?... Please…

**Caller:** Yeh.

**System:** Please rate your pain on a scale of 0 to 10, with 0 being no pain and 10 being the worst possible pain.

**Caller:** Four.

**System:** Was that 4?

**Caller:** Yeah.

**System:** Is the pain still in your left shoulder?

**Caller:** Yes.

**System:** Does it still feel sharp?

**Caller:** No.

**System:** How does the pain feel?

**Caller**: It’s dull.

**System**: Was that “dull?’’

**Caller:** Yeah.

**System:** Is the pain constant or does it come and go?

**Caller:** It’s constant.

**System:** Do you have any other symptoms?

**Caller:** No.

**System:** Would you like to leave a message for the staff?

**Caller:** No.

**System:** Thanks for your cooperation. I’ve got all the information that I need. I’ll call you again tomorrow. Goodbye.

**Table 2:** Automatically generated report for the follow-up session in Textbox 2

	**Captured Value**	**Confirmed (yes/no)**	**Confidence Score**
PIN	*****	no	74
Are you in pain?	yes	no	85
Medication taken?	yes	no	76
Medication time	9:45 am	yes	69
Medication helped?	yes	no	75
Pain rating	4	yes	87
Pain location	left shoulder	yes	87
Pain type	dull	yes	86

### Feasibility Study

Experimental evaluation of usability of the Pain Monitoring Voice Diary was performed with 24 volunteers (8 females, 16 males), mostly City College students. The volunteers were instructed either to refer to their past injury/sickness/pain episode experiences or to choose a scenario out of a set of nine that included scenarios for migraine pain, back pain, post-surgical pain (knee injury), arthritis and others. The goal of this evaluation was to prove the feasibility of data capture through dialogue and validate the assumptions underlying dialogue design.

The volunteers were asked to contribute 10 sessions with the system over a period of 2 weeks; in practice, the number of sessions per subject ranged from 1 to 20. There was no formal training session provided; instead, once enrolled (through a website) the subjects received an email notification with their PIN and general information about the system. The subjects were asked to either relate to pain episodes in their past while answering the system’s questions or use as a guidance one of nine provided medical scenarios compiled by a pain specialist, ranging from migraines and back pain to post-surgery pain (knee injury) and cancer- and chemotherapy-related afflictions.

## Results

### Dialogue Evaluation

From 24 volunteers we collected a total of 177 dialogue sessions: 171 sessions were completed, while the caller hung up in 6 sessions; 66 of the completed sessions were the follow-up type. There were a total of 2437 dialogue turns, where dialogue turn corresponds to one system prompt and one user utterance. The data capture rate, measuring the percentage of slots filled automatically, was 98%, while the other 2% were flagged for transcription. Data capture rate is not a direct measure of ASR accuracy since slots are not necessarily filled after the first attempt. Among the utterances sent to transcription, where the user had opted for the “none of those” option, 70% corresponded to the type of pain slot, 20% to the symptoms slot, and 10% to the body part slot, indicating that those are the grammars with the highest out-of-vocabulary rate. Since all captured slots were confirmed, to evaluate the accuracy of the captured data we had to evaluate the accuracy of the confirmation grammar used to recognize confirmation utterances. Among 859 confirmation utterances, 10 were misrecognized, leading to a 98.8% data accuracy rate. The rejection parameters in the grammar were tuned for an equal misrecognition/rejection rate and, indeed, the number of rejections among the 859 confirmation utterances was 11.

**Table 3:** Average dialogue session statistics (figures in parentheses are standard deviations)

Session duration (s)	99.34 (45.92)
Number of dialogue units per session	7.65 (2.48)
Duration of dialogue unit (s)	12.99 (2.7)
Dialogue turns per dialogue unit	1.86 (0.43)
Percentage of task-oriented turns	82% (15.4)
Percentage of interrupted prompts	68% (13)
Time duration of a dialogue turn (s)	6.97 (1.3)
Time duration of a dialogue turn when interruption was disabled	10.63 (1.5)

Table 3 shows other metrics [19] derived from dialogues. The high standard deviations of session duration and dialogue units per session are due to the extensive variability of dialogue sessions. The sessions not only differ by type (normal and follow-up), but there is also branching within the same type application (eg, some of the users report symptoms, while others don’t, some take medications). In addition, there is a great variability due to ASR errors and different possibilities inherent in the design of the call flow (eg, caller initiated help requests, speech recognition error handling such as re-prompts, negative confirmations).

The high standard deviations in caller utterances per dialogue unit and dialogue unit duration are due to the fact that not all dialogue units are created equal. For example, the “Are you in pain?” dialogue unit can fill a slot with a single “yes”/“no” utterance, while the pain location unit requires at least two dialogue utterances (body part and confirmation) if speech recognition does not fail, and more if it does.

Percentage of task-oriented dialogue turns (82%) (those dialogue turns that are *not* due to speech recognition errors or caller help requests) is a measure of dialogue efficiency: if there were no errors and help requests at all, it would be 100%. The prompts in the dialogue were designed to be interrupted by experienced callers. To quantify the use of interruption, we computed the percentage of interrupted prompts (68%). To quantify how far in the prompts the interruption occurs, we computed the average duration of dialogue turn (6.97 s), and compared it to the reference of average duration of dialogue turn (10.63 s) when the interruption feature was disabled.

### Evaluation of Flexible Level of User Support

One of the goals of the dialogue design described above was to have a flexible and adaptive user support for different types of users, providing short prompts and efficient call flow for experienced users and more detailed information in a troublesome situation for novice users. To evaluate the efficiency of the dialogues as a function of user proficiency, we divided the sessions into seven classes according to the sequential order of the session with the same user. Table 4 shows some statistics of the classes. For example, class A contains all the first sessions each of the 24 users had, with a total of 308 dialogue turns; class G contains all the sessions (whose ordinal number was 10 and above) for which the users had at least nine sessions previously completed.

**Table 4:** Dialogue sessions divided according to the call order

**Class Name**	**Call Order**	**Sessions**	**Turns**
A	1	24	308
B	2	19	302
C	3	15	206
D	4, 5	27	380
E	6, 7	23	324
F	8, 9	21	305
G	10+	43	612

**Figure 3:**
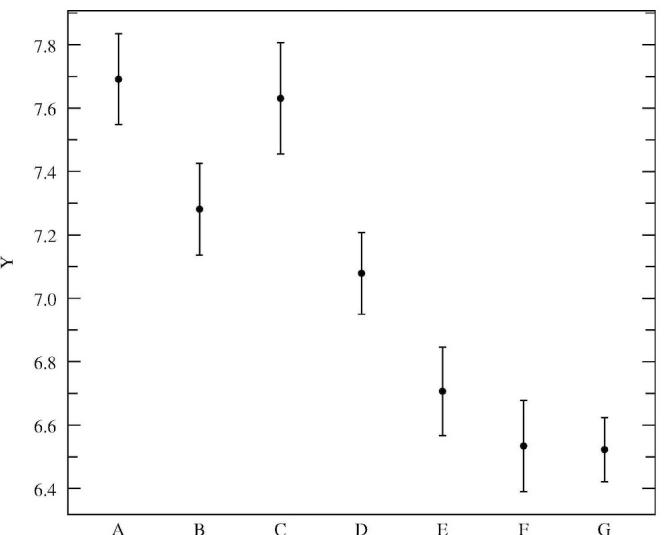
Average turn duration, in seconds, for dialogues in classes A to G (a dialogue turn corresponds to one system prompt and one user utterance; classes indicate increased experience, with A being the most inexperienced users; error bars indicate 95% confidence intervals)

**Figure 4:**
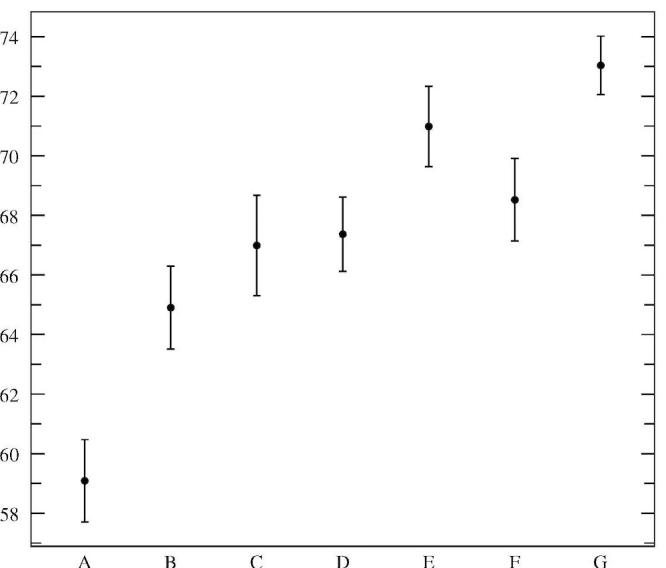
Percentage of interrupted prompts for dialogues in classes A to G (error bars indicate 95% confidence intervals)

**Figure 5:**
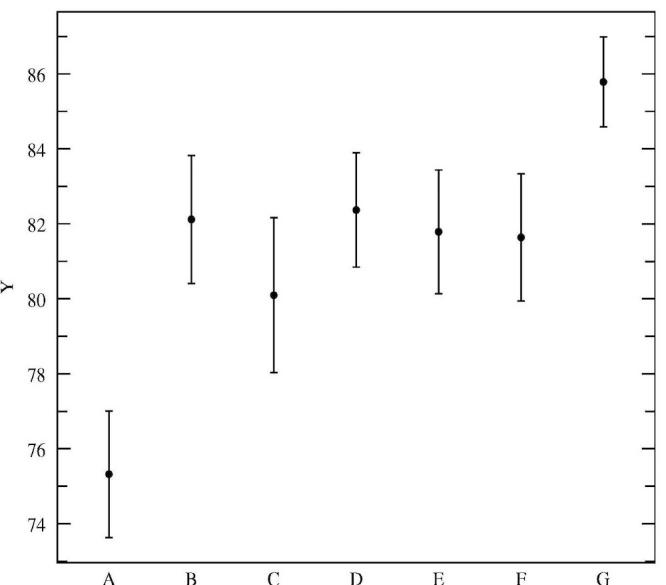
Percentage of task-oriented turns for dialogues in classes A to G (error bars indicate 95% confidence intervals)

Figures 3, 4, and 5 illustrate the average dialogue turn duration, average percentage of interrupted prompts, and average percentage of task-oriented prompts, respectively, for the classes outlined in Table 4. The differences between the seven session classes for the three metrics shown are statistically significant as tested by ANOVA [20] (F = 49.33, 50.40, and 50.40, respectively; df = 6; *P* < .001). The results in Figures 3 and 4 confirm the assumptions of the dialogue design: the prompts were designed to be interrupted by experienced users, and, indeed, the results indicate that the more experienced the user is, the more often and earlier she will interrupt. The novice user only interrupts in 59% of the prompts, with an average turn duration of 7.7 s, while users that had more than nine sessions completed interrupted in 73% of the prompts, with an average dialogue turn duration of 6.5 s. Figure 5 shows that with experience the users become more efficient with the system, as measured by the percentage of task-oriented dialogue turns: for novice users this percentage averages 75%, increasing to around 81% after just one previous session was completed, and up to 86% after at least nine sessions were previously completed.

## Discussion

In this paper we present a new methodology for real-time data collection of information from patients for health, behavioral, and lifestyle studies and monitoring.The voice data collection system is intended to facilitate real-time collection of information from patients via automated speech telephony delivery. A flexible self-report system gives patients the freedom to choose to use a phone as a device that meets their preferences, schedules, or limitations. The system monitors and tracks data collection compliance and generates real-time notifications and alerts for participants and administrators. The system provides on-demand/online reports to enhance informed decision making to improve patient care. The reports can be tailored to profile patient function over time and highlight clinically meaningful changes in health status.

Some of the major challenges in designing such a system include maintaining adequately high accuracy of the captured data while guaranteeing a satisfactory user experience. We described dialogue design for a Pain Monitoring Diary that targets these challenges. In particular, to control the accuracy of automatically captured data, we tuned the parameters of the garbage model–based confirmation grammar to reliably reject out-of vocabulary utterances; every captured value was explicitly confirmed using this high-accuracy confirmation grammar, and, if needed, hard to recognize or out-of-vocabulary answers were recorded and flagged for later transcription. To provide a flexible level of user support, we used a variety of methods, including designing the prompts to be interrupted by experienced users while carrying enough information for novices; providing the user with on-demand, context-dependent help; and detecting troublesome situations and guiding the user through them with more informative prompts or resorting to recording of the answer provided and flagging it for later transcription. The results of the feasibility study indicate that desired accuracy of data can be achieved with still a high degree of automation (98.8% data accuracy with 98% automation). The users were capable of using the flexible interface, with the sessions becoming more and more efficient as the users’ experience increased, both in terms of session duration and avoidance of troublesome dialogue situations. The adaptation data shown in Figures 3 to 5 suggest that even some rudimentary experience with the system increases the session efficiency significantly. While the subjects in the feasibility study did not receive any training or orientation session with the system, these results suggest that there could be significant value in conducting such sessions, which may provide users with the experience to jump start in the middle.

Finally, we would like to stress that one of the major weaknesses of the speech system is its single modality. Protocols that rely heavily on graphics and visual formats that cannot be completely replicated in speech are probably a poor match for this technology. Therefore, we do not see the spoken dialogue–based data collection as a replacement for existing data collection methodologies, but only as another choice for health care providers and researchers.

## Multimedia Appendix 1

Recording of a dialogue session with the system in normal mode (Transcription of this session appears in Textbox 1.)

## Multimedia Appendix 2

Recording of several dialogue exchanges with the system illustrating the rejection of out-of-vocabulary utterances

## Multimedia Appendix 3 and 4

Recordings of several dialogue exchanges illustrating the use of recording to capture out-of-vocabulary or problematic user inputs

## Multimedia Appendix 5-7

Recordings of different user experiences with the “Where does it hurt?” prompt

## Multimedia Appendix 8

Recording illustrating system behavior when help is requested

## Multimedia Appendix 9

Recording illustrating system behavior when it detects a problematic situation

## Multimedia Appendix 10

Recording of reminder style prompts in follow-up mode

## Multimedia Appendix 11

Recording of a dialogue session with the system in follow-up mode (Transcription of this session appears in Textbox 2; this session is the follow-up for the normal session in Textbox 1 and recorded in Multimedia Appendix 1.)

## Abbreviations

ASR: automated speech recognition
EMA: ecological momentary assessment
IVR: interactive voice response
PDA: personal digital assistant

## References

[1] Collins RL, Morsheimer ET, Shiffman S, Paty JA, Gnys M, Papandonatos GD Ecological momentary assessment in a behavioral drinking moderation training program.

[2] O'Connell K A, Gerkovich M M, Cook M R, Shiffman S, Hickcox M, Kakolewski K E (1998). Coping in real time: using Ecological Momentary Assessment techniques to assess coping with the urge to smoke. Res Nurs Health.

[3] Hufford MR, Shifford S (2000). Capturing real-time, real-world quality of life data using ecological momentary assessment. Quality of Life Newsletter.

[4] Turk DC, Melzack R (2001). Handbook of pain assessment. 2nd edition.

[5] Smyth J, Stone AA (2003). Ecological momentary assessment research in behavioral medicine. Journal of Happiness Studies.

[6] Smyth J, Wonderlich S, Crosby R, Miltenberger R, Mitchell J, Rorty M (2001). The use of ecological momentary assessment approaches in eating disorder research. Int J Eat Disord.

[7] Stone Arthur A, Shiffman Saul, Schwartz Joseph E, Broderick Joan E, Hufford Michael R (2002). Patient non-compliance with paper diaries. BMJ.

[8] Paul James, Seib Rachael, Prescott Todd (2005). The Internet and clinical trials: background, online resources, examples and issues. J Med Internet Res.

[9] Collins R Lorraine, Kashdan Todd B, Gollnisch Gemot (2003). The feasibility of using cellular phones to collect ecological momentary assessment data: application to alcohol consumption. Exp Clin Psychopharmacol.

[10] Black L, McTear M, Black N, Harper R, Lemon M The voice-logbook: integrating human factors for chronic care system.

[11] Black L, McTear M, Black N, Harper R, Lemon M Evaluating the DI@L-log system on a cohort of elderly, diabetic patients: results from a preliminary study.

[12] Levin E, Levin A Spoken dialog system for real-time data capture.

[13] Levin E, Levin A Dialog design for user adaptation.

[14] Davis Mellar P, Walsh Declan (2004). Cancer pain: how to measure the fifth vital sign. Cleve Clin J Med.

[15] Daut R L, Cleeland C S, Flanery R C (1983). Development of the Wisconsin Brief Pain Questionnaire to assess pain in cancer and other diseases. Pain.

[16] McTear M (2004). Spoken dialogue technology – towards the conversational user interface.

[17] Rose R, Paul D A hidden Markov model based keyword recognition system.

[18] Manos A, Zue V A segment-based spotter using phonetic filler models.

[19] Walker MA, Litman DJ, Kamm CA, Abella A (1997). PARADISE: a framework for evaluating spoken dialogue agents. Proceedings of ACL/EACL 35th Annual Meeting of the Association for Computational Linguistics.

[20] Edgington ES (1986). Randomization tests.

